# Lens Status Influences the Association between *CFH* Polymorphisms and Age-Related Macular Degeneration: Findings from Two Population-Based Studies in Singapore

**DOI:** 10.1371/journal.pone.0119570

**Published:** 2015-03-18

**Authors:** Chee Wai Wong, Jiemin Liao, Gemmy C. Cheung, Chiea Chuen Khor, Eranga N. Vithana, Jie Jin Wang, Paul Mitchell, Tin Aung, Tien Y. Wong, Ching-Yu Cheng

**Affiliations:** 1 Singapore Eye Research Institute, Singapore National Eye Centre, Singapore, Singapore; 2 Department of Ophthalmology, National University of Singapore and National University Health System, Singapore, Singapore; 3 Duke-NUS Graduate Medical School, Singapore, Singapore; 4 Division of Human Genetics, Genome Institute of Singapore, Singapore, Singapore; 5 Centre for Vision Research, Department of Ophthalmology, Westmead Millennium Institute, University of Sydney, Sydney, Australia

## Abstract

**Aims:**

To determine the differential effects of genetic polymorphism in *CFH* and *ARMS2* on risk of age-related macular degeneration (AMD) between phakic vs. pseudophakic/aphakic eyes.

**Methods:**

9,529 eyes of 4,918 participants from the Singapore Malay Eye Study and Singapore Indian Eye Study were analyzed. Participants had detailed eye examinations, including slit-lamp examinations and dilated fundus photography. AMD grading was performed according to the Wisconsin age-related maculopathy grading system. Lens status was defined. Single nucleotide polymorphisms (SNPs) rs10801555 (Y402H) within *CFH* and rs3750847 in *ARMS2* were assessed. The main outcome measure was early AMD or any AMD.

**Results:**

No significant associations between the *CFH* Y402H genotypes and early AMD were found in phakic individuals. In contrast, among pseudophakic/aphakic individuals, the *CFH* Y402H risk genotypes were significantly associated with higher odds of early AMD, with an OR of 1.57 (95% CI: 1.07–2.29) for GA genotype and 2.40 (95% CI: 1.25–4.61) for AA genotype, compared to those with GG genotype. There was significant interaction between pseudophakic/aphakic status and *CFH* Y402H variant on risk of early AMD (p = 0.037), adjusting for age, gender, and the first 5 genetic principal components. No significant interaction was found between lens status and *ARMS2* rs3750847.

**Conclusions:**

*CFH* genetic polymorphism and pseudophakic/aphakic status may have a potential synergistic effect on early AMD, suggesting roles for the complement system and related pathways in the pathogenesis of AMD in eyes after cataract surgery.

## Introduction

Age-related macular degeneration (AMD) is the leading cause of irreversible blindness in both developed and developing countries [1–5]. AMD is a pathogenically complex disease, an outcome of both multiple environmental and genetic factors. Much research has been undertaken to examine individual environmental and genetic risk factors for early and late AMD [6–8].

Regarding genetic factors, the *CFH* Y402H on chromosome 1q32 was the first identified AMD susceptibility variant [9–11]. Subsequently, *ARMS2/HTRA1* on chromosome 10q26 was also found to be associated with risk of AMD [12,13]. *ARMS2* risk alleles were found to be preferentially associated with risk of neovascular AMD while *CFH* risk alleles were more frequently associated with risk of geographic atrophy [14,15]. A recent meta-analysis of genome-wide association studies including >17,000 advanced AMD cases and >60,000 controls confirmed the associations at *CFH* (odds ratio [OR] = 2.43) and *ARMS2* (OR = 2.76) loci [16]. Other variants associated with AMD have much smaller effect sizes, and include genes involved in complement system, cholesterol metabolism, and angiogenesis pathways [17]. In Asians, however, the relationship and contribution of these genetic risk loci on AMD are less clear with fewer studies available [18].

Besides smoking, cataract surgery is the most consistent “environmental” risk factor for AMD observed in previous studies [7,19,20], including a meta-analysis of three prospective studies in Caucasian populations [20–22]. In Asians, the relationship of cataract surgery and AMD is again less clear. The Beijing Eye Study for example did not find an association of either early or late AMD with cataract surgery in Chinese persons [23]. Furthermore, the exact mechanism for the increased risk of AMD after cataract surgery remains unknown and may involve increased exposure to blue visible light [24,25] or a chronic foreign body inflammatory response in the eye due to implantation of an intraocular lens [26].

Genetic interactions with environmental factors provide important insights into the understanding of the pathophysiology of AMD. We hypothesized that persons with the two major genetic polymorphisms would have a higher risk of AMD and the risk was aggravated in eyes with past cataract surgery. We therefore conducted this study to assess the independent associations of *CFH* and *ARMS2* genetic polymorphisms and lens status (i.e., pseudophakic/aphakic eyes) with AMD, and to investigate the gene-environment interaction between lens status and genetic polymorphisms on the risk of this disease.

## Methods

### Study population

Our study population consisted of subjects from 2 population-based studies: the Singapore Malay Eye Study (SiMES) (2004–2006) and the Singapore Indian Eye Study (2007–2009). The detailed methodologies of these studies have been published elsewhere [27,28]. In brief, an age-stratified random sampling was used to select ethnic Malays and Indians 40 to 80 years of age, who were living in Singapore during each stipulated study period. These studies were conducted at the Singapore Eye Research Institute with approval from the Singhealth Institutional Review Board. The study was conducted in accordance with the Declaration of Helsinki, with written informed consent obtained from all subjects before participation.

### Clinical examination

Standardized systemic and ocular examinations, interviewer-administered questionnaires, and standard blood investigations were conducted on the same day for all participants.

A digital nonmydriatic retinal camera (Canon CR-DGi with a 10D SLR backing, Canon, Japan) was used to obtain color photographs of Early Treatment for Diabetic Retinopathy Study (ETDRS) standard field 1 (centered on the optic disc) and ETDRS standard field 2 (centered on the fovea) of each eye. The photographs were saved to hard disk, backed up on DVDs, and sent for grading for the presence of AMD using the Wisconsin age-related maculopathy grading system [29]. Standardized slit-lamp examinations (Haag-Streit model BQ-900; Haag-Streit, Bern, Switzerland) were performed by trained study ophthalmologists after pupil dilatation to determine lens status (phakic, pseudophakic or aphakic) and the fundus was examined with a 78D lens.

A detailed interviewer-administered questionnaire was used to collect relevant sociodemographic data and medical history from all participants. Data such as education, occupation (predominantly indoor or outdoor), lifestyle risk factors (e.g., smoking and alcohol intake), and medical and surgical histories were collected. Blood pressure was measured with a digital automatic blood pressure monitor (Dinamap model Pro Series DP110X-RW, 100V2; GE Medical Systems Information Technologies, Inc, Milwaukee, WI) after the participants were seated for at least 5 minutes. Nonfasting venous blood samples were collected for biochemistry tests, including serum lipids (total cholesterol, high-density lipoprotein cholesterol, low-density lipoprotein cholesterol), glycosylated hemoglobin A1c, creatinine, and random glucose. All cholesterol levels were analyzed in participants on a continuous scale. The average of the 2 systolic and diastolic blood pressure measurements was used as the systolic and diastolic blood pressure value. Hypertension was defined as systolic blood pressure of 140 mm Hg or more or diastolic blood pressure of 90 mm Hg or more or self-reported physician-diagnosed hypertension. Diabetes mellitus was defined as random glucose of 11.1 mmol/L or more, use of diabetic medication, or a physician diagnosis of diabetes mellitus. Hyperlipidemia was defined as a total cholesterol level of 239.4 mg/dL or more (to convert to mmol/L multiply by 0.0259), or use of lipid lowering medication. Chronic kidney disease was defined as an estimated glomerular filtration rate of less than 60 mL/min per 1.73 m^2^ based on the Chronic Kidney Disease Epidemiology Collaboration (CKD-EPI) equations [30].

### AMD grading

Photograph grading was performed in a standard manner according to the Wisconsin age-related maculopathy grading system. The prevalence of AMD in SiMES and the Singapore Indian Eye Study was 7.7% and 5.7%, respectively [31,32]. Among the AMD features evaluated were drusen size, type, and area; increased retinal pigment; retinal pigment epithelial depigmentation; pure geographic atrophy; and signs of exudative macular degeneration. Drusen were classified as hard or soft; then soft drusen were divided into distinct and indistinct soft drusen. Early AMD was defined by either any soft drusen (distinct or indistinct) and pigmentary abnormalities or large soft drusen 125 μm or more in diameter with a large drusen area (>500-μm-diameter circle) or large soft indistinct drusen in the absence of signs of late AMD. Late AMD was defined by the presence of any of the following: geographic atrophy or pigment epithelial detachment, subretinal hemorrhage or a visible subretinal new vessel, or a subretinal fibrous scar or laser treatment scar for AMD.

### Genotyping

All samples were genotyped using Illumina Human610-Quad BeadChips (Illumina Inc.), which assays 620,901 SNPs across the genome, according to manufacturer protocols. In brief, quality control (QC) criteria included a first round for autosomes SNP QC to obtain a cleaned set of genotypes for sample QC, by excluding SNPs with: (1) missingness (per SNP call rate) >5%; (2) minor allele frequency (MAF) < 1%; and (3) Hardy Weinberg Equilibrium (HWE) p-value <10^−6^. Using the subset of SNPs passing the first round QC, samples were then excluded based on the following conditions: (1) per-sample call rates of less than 95%; (2) excessive heterozygosity (defined as the sample heterozygosity); (3) cryptic relatedness; (4) gender discrepancies; and (5) deviation in population membership from population structure analysis. A second round of SNP QC was then applied to the remaining samples passing quality checks. We excluded SNPs with missingness >5%, gross departure from HWE (p <10^−6^) and MAF<1%. The commonly associated SNPs in the *CFH* and *ARMS2/HTRA-1* genes, namely rs1061170 and rs10490924, were not included in the Human610-Quad BeadChips. Hence, we proceeded to test other SNPs within the genes of interest i.e. *CFH* and *ARMS2/HTRA-1* for associations with AMD as well as interaction effects with lens status on the association with AMD. We chose the SNP rs10801555 for CFH and rs3750847 for ARMS2 after confirming that they were in perfect linkage disequilibrium (LD; r^2^ = 1.0) with the Y402H variant (rs1061170) for *CFH* and rs10490924 for *ARMS2* [33]. In SiMES and SINDI, the MAF for rs10801555 is 0.09 and 0.28, respectively, and the MAF for rs3750847 is 0.39 and 0.34, respectively.

### Statistical Analysis

Statistical analysis was performed with STATA version 12.0 (Texas, USA). A p value of <0.05 was considered statistically significant. Comparison of groups was performed with chi square tests for categorical variables, and two sample t-tests for continuous variables. In multivariate analysis of the main effect of pseudophakic/aphakic status, the OR (95% confidence interval [CI]) of AMD presence was adjusted for potential risk factors, including age, gender, smoking status, ethnicity, hypertension, diabetes, hyperlipidemia and chronic kidney disease. Data from both eyes of the same individual were included in the analysis, and generalized estimating equation (GEE) analysis [34] was used to account for correlation between eyes from the same individual.

For gene-environmental (G x E) interaction, we performed a 1 df test for β_GE_ using the GEE model:

$$\text{logit P}\left(\text{AMD}=1|X,\text{G,E}\right)=\beta_{0}+\beta_{X}X+\beta_{\text{G}}\text{G}+\beta_{\text{E}}\text{E }+\beta_{\text{GE}}\text{GE}$$

where **X** is a matrix of covariates as listed in the main effect model; G is the number of risk allele for a SNP (rs10801555: A; rs3750847: T), coded as 0, 1 or 2; E is the environmental exposure status, whether the individual had pseudophakic/aphakic eyes. The p-value of the interaction term β_GE_ GE was used to assess the significance of the interaction between lens status and genetic variants. Odds ratios and their 95% CIs were calculated per copy of risk allele, assuming a log-additive increase for each risk allele. To control for population substructure in our study population, we performed principal component (PC) analysis using the EIGENSTRAT software v4.2 [35], and the first 5 PCs, were included as covariates in our regression models.

## Results

There were 6,680 participants from SiMES and SINDI. 3,236 eyes were excluded as those participants either were not genotyped or failed genotyping QC. A further 595 eyes were excluded due to incomplete data for AMD and lens status. A total of 4,918 subjects with 9,529 eyes were left and included in the study. Of these, 4,799 subjects have complete data on the following variables: early AMD, any AMD, gender, smoking, age, hypertension, diabetes, hyperlipidaemia, CKD, *CFH* rs10801555 and *ARMS2* rs3750847. **Table 1** shows the characteristics of subjects without AMD, with early AMD and with any AMD. There were 4,626 subjects without AMD, 274 subjects with early AMD and 292 subjects with any AMD. AMD cases were more likely to be older men, with hypertension, hyperlipidemia and chronic kidney disease (all p <0.05). Similar results were obtained for any AMD cases.

**Table 1:** Characteristics of subjects without AMD, with early AMD and with any AMD.

Demographics	All (N = 4918)^a^	Without AMD (N = 4626)	With Early AMD (N = 274)	With Any AMD (N = 292)	P Value^b^	P Value^c^
Age (yrs)	58.54 (10.45)	58.07 (10.28)	66.02 (10.31)	66.1 (10.27)	<0.001	<0.001
Female	2441 (49.63)	2332 (50.41)	105 (38.32)	109 (37.33)	<0.001	<0.001
Current smokers	872 (17.76)	828 (17.92)	39 (14.34)	44 (15.17)	0.133	0.235
Hypertension	3097 (63.23)	2863 (62.16)	217 (79.20)	234 (80.14)	<0.001	<0.001
Diabetes	1395 (28.69)	1304 (28.51)	86 (31.73)	91 (31.49)	0.254	0.278
Hyperlipidaemia	2119 (43.63)	1976 (43.25)	134 (49.63)	143 (49.65)	0.040	0.034
Chronic kidney disease	761 (15.48)	688 (14.88)	69 (25.18)	73 (25.00)	<0.001	<0.001
*CFH* rs10801555						
GG	3333 (67.77)	3144 (67.96)	180 (65.59)	189 (64.73)		
GA	1357 (27.59)	1276 (27.58)	75 (27.37)	81 (27.74)	0.160	0.049
AA	228 (4.64)	206 (4.45)	19 (6.93)	22 (7.53)		
*ARMS2* rs3750847						
CC	1992 (40.50)	1885 (40.75)	103 (37.59)	107 (36.53)		
CT	2257 (45.89)	2133 (46.11)	111 (40.51)	124 (42.47)	<0.001	<0.001
TT	669 (13.60)	608 (13.14)	60 (21.90)	61 (20.89)		

Data presented are no. (%), except for age, which is presented as mean (SD). Analysis was done by individual level.

^a^ Number of subjects with missing data for the following variables: current smokers, 8; hypertension, 20; diabetes, 55; hyperlipidaemia, 61; chronic kidney disease, 1.

^b^ For comparison between individuals without AMD vs with early AMD, using chi-square tests for categorical variables, and two sample t-tests for continuous variables.

^c^ For comparison between individuals without AMD vs with any AMD, using chi-square tests for categorical variables, and two sample t-tests for continuous variables.

Of the 9,529 eyes, 925 (9.7%) were either pseudophakic or aphakic (**Table 2**). More Indian subjects were pseudophakic/aphakic than Malays (p <0.001). There were no differences in distribution of early or any AMD between Malays and Indians (**Table 2).**

**Table 2:** Distribution of AMD and cataract surgeries by ethnicity.

	Total (N = 9529 Eyes)	Malays (N = 4675 Eyes)	Indians (N = 4854 Eyes)	P Value^a^
Early AMD	346 (3.63)	159 (3.41)	187 (3.86)	0.243
Late AMD	31 (0.33)	18 (0.40)	13 (0.28)	0.321
Any AMD	377 (3.96)	177 (3.79)	200 (4.12)	0.403
Past Cataract Surgery				
Pseudophakic	916 (9.62)	329 (7.04)	587 (12.10)	
Aphakic	9 (0.09)	5 (0.11)	4 (0.08)	
Total	925 (9.71)	334 (7.14)	591 (12.18)	<0.001

Data presented are no. (%). Analysis was done on eye level.

^a^ *P* values are for 2x2 comparisons between variable of interest (yes/no) and ethnicity (malays/indians), 1 degree of freedom chi-square test. Pseudophakic/aphakic eyes were more likely have early AMD (9.1%) or any AMD (9.3%), compared to phakic eyes (3.0% and 3.4% with early and any AMD, respectively, both p <0.001, Table 3). In the univariate analysis, the *ARMS2* (rs3750847) genotype was significantly associated with early AMD and any AMD (both p <0.001), while the *CFH* (rs10801555) genotype had marginally significant association with any AMD (p = 0.049) but not with early AMD (p = 0.160, Table 1).

**Table 3:** Distribution of AMD by presence or absence of previous cataract surgery.

	Pseudophakic/Aphakic Eyes (N = 925)	Phakic Eyes (N = 8604)	P Value^a^
Early AMD	84 (9.1)	262 (3.0)	<0.001
Any AMD	86 (9.3)	291 (3.4)	<0.001

Data presented are no. (%). Analysis was done on eye level.

^a^ *P* values are for 2x2 comparisons between pseudophakic/aphakic eyes vs. phakic eyes, 1 degree of freedom chi-square test.

**Table 4** shows the assessment of each main effect in isolation (lens status or risk genotypes). After adjusting for age, gender, ethnicity, smoking status, hypertension, diabetes, hyperlipidemia, chronic kidney disease, pseudophakic/aphakic eyes remained more likely to have early AMD (OR = 1.36; 95% CI: 0.99–1.87) or any AMD (OR = 1.21, 95% CI: 0.89–1.66), although these associations did not reach statistical significance (Model 2 in **Table 4**). Consistent with the univariate analysis, the *ARMS2* SNP was associated with early AMD in the models adjusted for potential risk factors (Model 1, p = 0.002; Model 2, p = 0.001, **Table 4**) and any AMD (Model 1, p = 0.001; Model 2, p <0.001, **Table 4**).

**Table 4:** Multivariate analysis showing the association of AMD with lens status, and the *CFH* and *ARMS*2 SNPs.

		Early	AMD			Any	AMD	
	Model 1^a^ (N = 9498)		Model 2^b^ (N = 9269)		Model 1^a^ (N = 9529)		Model 2^b^ (N = 9300)	
	OR (95% CI)	P Value	OR (95% CI)	P Value	OR (95% CI)	P Value	OR (95% CI)	P Value
Lens status								
Phakic	[Reference]		[Reference]		[Reference]		[Reference]	
Pseudophakic/Aphakic	1.29 (0.95–1.77)	0.108	1.36 (0.99–1.87)	0.058	1.16 (0.85–1.57)	0.349	1.21 (0.89–1.66)	0.225
*CFH* rs10801555								
GG	[Reference]		[Reference]		[Reference]		[Reference]	
GA	1.10 (0.88–1.37)	0.421	1.09 (0.86–1.37)	0.478	1.17 (0.94–1.45)	0.160	1.16 (0.93–1.44)	0.187
AA	1.20 (0.77–1.88)		1.18 (0.75–1.87)		1.36 (0.89–2.09)		1.34 (0.87–2.08)	
*ARMS2* rs3750847								
CC	[Reference]		[Reference]		[Reference]		[Reference]	
CT	1.32 (1.11–1.58)	0.002	1.37 (1.14–1.64)	0.001	1.33 (1.12–1.58)	0.001	1.37 (1.15–1.63)	<0.001
TT	1.75 (1.23–2.51)		1.86 (1.30–2.68)		1.77 (1.25–2.50)		1.88 (1.32–2.67)	

OR, odds ratio; CI, confidence interval.

^a^Multivariate logistic regression using general estimating equations, adjusted for age, gender, and ethnicity for the effect of lens status, and additionally adjusted for the first 5 genetic principal components for the effects of SNPs.

^b^Adjusted for the covariates in Model 1 and current smoker, hypertension, diabetes, hyperlipidaemia, and chronic kidney disease.

**Fig 1:**
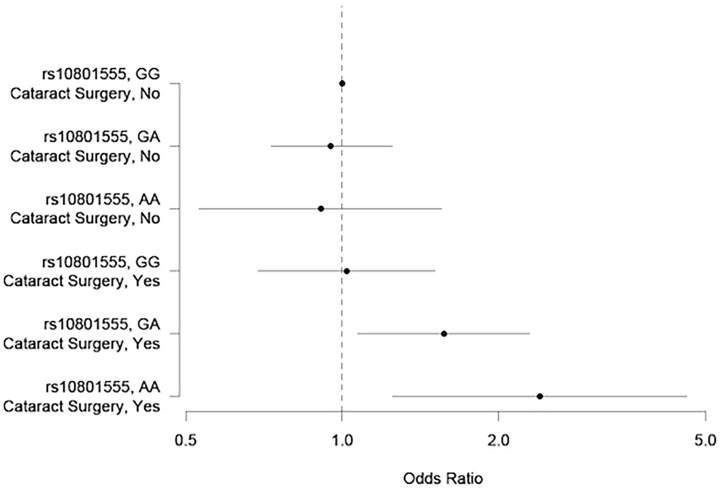
Forest plot of joint effects of pseudophakic\aphakic status and *CFH* SNP on risk of early AMD.

Stratified analysis was performed for the phakic vs. pseudophakic/aphakic groups (**Table 5),** adjusted for age, gender and the first 5 genetic principal components. In phakic eyes, the risk of early AMD was similar in those with the *CFH* Y402H risk genotypes (genotype GA: OR = 0.95, 95% CI: 0.73–1.25; AA: OR = 0.91, 95% CI: 0.53–1.55), to those with GG genotype. In contrast, pseudophakic/aphakic eyes that had the Y402H risk genotype were more likely to have early AMD, OR 1.57 (95% CI: 1.07–2.29) for the GA genotype and OR 2.40 (95% CI: 1.25–4.61) for the AA genotype, compared to those that had the GG genotype and were phakic (**Fig. 1**). The interaction between pseudophakic/aphakic status and *CFH* Y402H on the risk of early AMD was significant (p = 0.037). After additional adjustment for potential confounders, namely smoking status, hypertension, diabetes, hyperlipidemia and chronic kidney disease, the interaction remained significant (p = 0.045). A similar association trend was observed for any AMD (**Table 5**), but the interaction did not reach statistical significance (p = 0.115). We performed the same analysis with another *CFH* SNP rs379489 and obtained similar results (p for interaction 0.015 for early AMD and 0.046 for any AMD). After removing aphakic eyes, the p value for interaction between early AMD and *CFH* remained significant (0.042), with minimal changes to OR or 95% CI. There was no significant difference in visual acuity between pseudophakic patients with or without early/any AMD who have at least 1 *CFH* risk allele (p = 0.175, data not shown).

**Table 5:** Joint effects of lens status and *CFH/ARMS2* SNP on risk of early AMD and any AMD.

	Early AMD,	OR^a^ (95% CI)	Any AMD,	OR^a^ (95% CI)
	Phakic Eyes (N = 8,575)	Pseudophakic/Aphakic Eyes (N = 923)	Phakic Eyes (N = 8,604)	Pseudophakic/Aphakic Eyes (N = 925)
*CFH* rs10801555				
GG	[Reference]	1.02 (0.69–1.51)	[Reference]	0.97 (0.66–1.42)
GA	0.95 (0.73–1.25)	1.57 (1.07–2.29)^b^	1.06 (0.82–1.36)	1.46 (1.00–2.12)
AA	0.91 (0.53–1.55)	2.40 (1.25–4.61)^c^	1.12 (0.68–1.85)	2.19 (1.14–4.20) ^b^
P value for interaction^d^		0.037		0.115
*ARMS2* rs3750847				
CC	[Reference]	1.15 (0.72–1.82)	[Reference]	1.05 (0.67–1.66)
CT	1.28 (1.04–1.57)^b^	1.69 (1.18–2.42)^c^	1.30 (1.07–1.58)^c^	1.53 (1.08–2.18) ^b^
TT	1.64 (1.09–2.46)^b^	2.48 (1.44–4.27)^c^	1.68 (1.14–2.49)^c^	2.23 (1.30–3.81) ^c^
P value for interaction^d^		0.495		0.570

OR, odds ratio; CI, confidence interval.

^a^ Odds ratios were calculated using multivariate logistic regression using general estimating equations, adjusted for age, gender and the first 5 genetic principal components.

^b^ *P* <0.05

^c^ *P* <0.01

^d^ *P*-value of the interaction term β_GE_ GE was used to assess the significance of the interaction between lens status and genetic variants.

For the *ARMS2* genotype, compared to phakic eyes with the CC genotype, the risk of having early AMD was higher both in pseudophakic/aphakic eyes (genotype CT: OR = 1.69, 95% CI: 1.18–2.42; TT: OR = 2.48, 95% CI: 1.44–4.27) and phakic eyes (genotype CT: OR = 1.28, 95% CI: 1.04–1.57; TT: OR = 1.64, 95% CI: 1.09–2.46). There was no significant interaction between lens status and the *ARMS2* SNP for either early AMD (p = 0.495) or any AMD (p = 0.570).

## Discussion

We identified a statistically significant interaction between lens status and the *CFH* gene polymorphism on the risk of early AMD, suggesting a possible synergistic effect between the two. Pseudophakic/aphakic status was found to increase the risk of early AMD in patients with *CFH* risk genotypes above the risk conferred by either factor alone.

There are few comparable studies. The Rotterdam Eye Study previously examined the incidence of AMD in patients with prior cataract who were *CFH* Y402H carriers, and found that the OR for dry AMD in *CFH* carriers after cataract surgery increased in an allele-dose manner with an OR of 2.30 (95% CI: 0.28–18.83) for non-carriers, 3.31 (95% CI: 1.23–8.91) for heterozygotes, and 4.02 (95% CI: 1.37–11.79) for homozygous CFH Y402H carriers [36]. Whether this increased risk was entirely due to *CFH* genetic susceptibility or represented an interaction effect was not explored. As far as we know, our study is the first to report a significant gene-environment interaction between CFH genetic polymorphism and lens status on the risk of AMD.

Importantly, in our study neither pseudophakic/aphakic status nor the *CFH* risk genotype alone had a significant association with AMD. An association was found only when both factors were present. This suggests an unmasking effect: genetic susceptibility leading to disease being more prominent in the presence of an environmental trigger or another susceptibility gene. This is exemplified in other conditions. For example, in phenylketonuria, a mutation in the gene for a hepatic enzyme phenylalanine hydroxylase (genetic susceptibility) results in disease only in the presence of a diet consisting of the amino acid phenylalanine (environmental trigger). In Alzheimer’s disease, *BACE1* polymorphism (susceptibility gene) alone was not associated with disease, but in the presence of another gene, *APOE* epsilon 4 (genetic trigger), the effect of *BACE1* was revealed [37]. This effect may, in part, explain why risk factors for disease appear to differ between populations. Genetic, environmental factors and their interactions differ between populations, some of which are unknown or unexplored, confounding the associations of known risk factors with AMD.

Our findings suggest that the complement pathway plays a role in the pathogenesis of AMD in pseudophakic/aphakic eyes. At least two mechanisms have been postulated for how *CFH* Y402H polymorphism may result in AMD. First, it may reduce binding of CRP to the CFH protein, affecting its ability to inhibit complement pathway. Second, it may affect the binding of CFH to cell surface glycosaminogycans (GAGs), thus limiting the function of CFH [38,39]. Cataract surgery with implantation of an intraocular lens (IOL) can cause inflammation in several ways. Older studies on uveal biocompatibility of IOLs have found epithelioid and giant cells on certain IOL materials, characteristics of a foreign body cell reaction [40–42]. Moreover, surgical trauma during cataract operation compromises the blood ocular barrier [43] and could expose the intraocular environment to circulating systemic mediators of inflammation. Lastly, Pokidysheva has shown in vitro activation of complement by an older type of intraocular lens, although in vivo correlation has not been demonstrated in animals or humans [44].

We simultaneously assessed the interaction between *ARMS2* and lens status on the risk of early AMD. Animal studies have demonstrated the role of *ARMS2* in the phagocytosis of photoreceptor outer segments [45]. In aphakic eyes or pseudophakic eyes that were not implanted with blue-light filtering (400–480nm) IOLs, the retina is exposed to UV light and blue visible light that would otherwise have been absorbed by the crystalline lens [46]. A possible effect of this exposure is an increase in generation of reactive oxygen species, which, coupled with lipofuscin accumulation from impaired RPE phagocytosis, may lead to eventual RPE cell death [24,47–49]. In our study, although the *ARMS2* SNP is associated with early AMD, lens status and the *ARMS2* genotypes did not have a significant synergistic effect. However, we observed that the ORs of having early AMD or any AMD were in general higher in pseudophakic/aphakic individuals, compared to those without past cataract surgery. Further studies are needed to conclude whether a synergistic effect exists.

The strengths of our study lie in the large number of individuals analyzed. As a population-based study, selection bias associated with hospital-based studies is minimized. However, our study is limited by the small number of patients with late AMD, precluding sufficient statistical power in this group. There may be potential residual confounding by a range of factors including amount of sun exposure and type of IOL implant used in the pseudophakic group, although none of these have been shown to be definitively associated with AMD. In addition, the differences in MAF of rs10801555 between Malays and Indians could potentially be a source of confounding, although this may have been accounted for to a certain extent by the use of principal components in our multivariate model. As with all cross-sectional observational studies, the lack of data on the temporal sequence of AMD development after cataract surgery hindered us from assessing causal relationships. Although we did not find significant differences in visual acuity between pseudophakic patients with or without early/any AMD who have at least 1 CFH risk allele, we did not have data on contrast sensitivity, which is more likely (than visual acuity) to be affected in patients with early stages of AMD. In addition, early AMD is a known risk factor for late AMD, which has significant impact on visual acuity. In view of these limitations, the clinical relevance of our findings is uncertain at this point in time but as the first study of its kind, our study provides a basis for future longitudinal cohort studies, to assess this synergistic effect, which, as discussed above, has potentially clinically significant applications.

In summary, based on our population-based studies, there is a possible synergistic interaction between *CFH* risk genotypes and lens status. Pseudophakic/aphakic eyes may have an increased risk of early AMD in patients with *CFH* risk genotypes over and above the risk conferred by either factor alone. Further longitudinal studies are needed to determine if the synergistic interaction between *CFH* risk genotypes and cataract surgery results in a higher incidence of AMD. Further research may be undertaken in IOL biocompatibility and other potential mechanisms to minimize complement activation in the eye after cataract surgery.
